# Lead (Pb) bioaccumulation and antioxidative responses in *Tetraena qataranse*

**DOI:** 10.1038/s41598-020-73621-z

**Published:** 2020-10-13

**Authors:** Kamal Usman, Mohammed H. Abu-Dieyeh, Nabil Zouari, Mohammad A. Al-Ghouti

**Affiliations:** Department of Biological and Environmental Sciences, College of Arts and Sciences, Qatar University, P O Box 2713, Doha, Qatar

**Keywords:** Abiotic, Ecophysiology

## Abstract

Lead (Pb) is the second most toxic metal on Earth and is toxic to humans and other living things. In plants, Pb commonly inhibits growth when it is at a concentration in the soil of 30 mg/kg or more but several Pb tolerant plants have been reported. However, few studies have focused on plant response to Pb exposure, particularly at concentrations higher than 30 mg/kg. The assessment and evaluation of metal dose-dependent plant responses will assist in future phytoremediation studies. Therefore, this work documents the Pb concentration-dependent antioxidative response in *Tetraena qataranse.* Young seedlings were irrigated with 0, 25, 50, and 100 mg/L Pb every 48 h for seven weeks under greenhouse conditions. A phytotoxicity test showed that at the lowest treatment concentration, Pb stimulates growth. However, at 100 mg/L (1600 mg/kg Pb in the growth medium at harvest), the metal disrupted healthy growth in *T. qataranse*, particularly root development. Metal accumulation in the root was higher (up to 2784 mg/kg) than that of the shoot (1141.6 mg/kg). Activity assays of superoxide dismutase (SOD), catalase (CAT), ascorbate peroxidase (APX), guaiacol peroxidase (GPX), and glutathione reductase (GR) showed a progressive increase in enzymatic activities due to Pb treatment. Together, the results of this study suggest that *T. qataranse* is a Pb hyperaccumulator. Increased antioxidant enzyme activity was essential to maintaining cellular homeostasis and assisted in the arid plant’s tolerance to Pb stress.

## Introduction

Environmental pollution is one of the most challenging sustainable development agendas today. Toxic metals, including Pb, are some pollutants that are most dangerous to human health. This is especially true in countries witnessing rapid industrialization^1,2^. In 2015, Pb was listed as the number one heavy metal on Earth. It is generally toxic to most plants at a soil concentration higher than 30 mg/kg^4^ .

The state of Qatar is located in the Arabian Peninsula desert and has a growing human population and increased anthropogenic activities. Evidence of the occurrence of some environmental pollutants, including toxic metals, has been documented. However, only a handful of studies have investigated the phytoremediation of toxic metals, including Pb, using native and invasive plants or plant responses to metal toxicity for optimized remediation^3–6^. Our group was among the first to conduct an assessment of the capacity of various local plants to accumulate different toxic metals, including Pb, under field conditions^7^. Though metal concentrations in the soil were relatively low, our study found *Tetraena qataranse* to be the most promising for phytoremediation. While the use of plants to remove toxic metals dates back decades and hundreds of plants have been reported thus far, climatic conditions and soil environments differ between regions and countries around the world^1,5^. Arid and semi-arid climates are known to be harsh for the majority of plant species.

Further, the scale-up of such a promising strategy still needs to be achieved, partly due to a poor understanding of the mechanism governing the toxic metal phytoremediation process. Studying promising plant's responses to toxic metal exposure will help in overcoming such a limitation. In this regard, high metal accumulation in plant tissues provides the perfect condition for evaluating such responses. To date, several Pb accumulating and tolerant plants have been reported. However, only a few hyperaccumulators exist. Additionally, most studies noted that heavy metals, like Pb, preferentially accumulate in roots compared to other plant parts^11,14,20,35^.

Several physiological, biochemical, and molecular mechanisms enable plants toxic metals tolerance. Transporters, and metal-binding proteins such as phytochelatins (PCs), and metallothioneins (MTs) are most notable in aiding toxic metal sequestration in plants^8^. PCs are induced by phytochelatins synthase (PCS), an enzyme that is triggered when metal ions are present^26^. MTs are low molecular weight gene-encoded, cysteine-rich proteins. Numerous studies sugeest that these molecules play important roles in Pb sequestration^2,20,26^. From antioxidative system perspectives, toxic metals, including Pb, interferes with cellular homeostasis by generating reactive oxygen species (ROS) in plant system. This negatively affects plant health, and consequently, may lead to death^8^. A more recent discussion on Pb accumulation and suggested tolerance and detoxification mechanisms can be found in a crical review by Kumar and Prasad^13^.

The antioxidant system is essential for shielding plants against heavy metal toxicity and does so by creating changes in the activities of critical enzymes including catalase (CAT), superoxide dismutase (SOD), ascorbate peroxidase (APX), glutathione reductase (GR), and guaiacol peroxidase (GPX)^6^. SOD is the first line of defense; it sequesters noxious superoxide ions and breaks them into less harmful hydrogen peroxide (H_2_O_2_) and oxygen molecules (O_2_). CAT assists in further detoxification by converting H_2_O_2_ into water (H_2_O) and oxygen molecules (O_2_). GR combats oxidative stress by balancing reduced (GSH) and oxidized glutathione (GSSG)^9,10^. Following heavy metal (e.g. Pb) exposure and uptake, determining the activities of antioxidative enzymes is vital to understanding plant response to metal toxicity from a biochemical and, in part, molecular perspective. Therefore, this study aims to further explore *T. qataranse* Pb tolerance by investigating its antioxidative response to varying concentrations (0, 25, 50, and 100 mg/L) of Pb under greenhouse conditions.

## Results

### Effect of Pb on seedling growth

**Figure 1:**
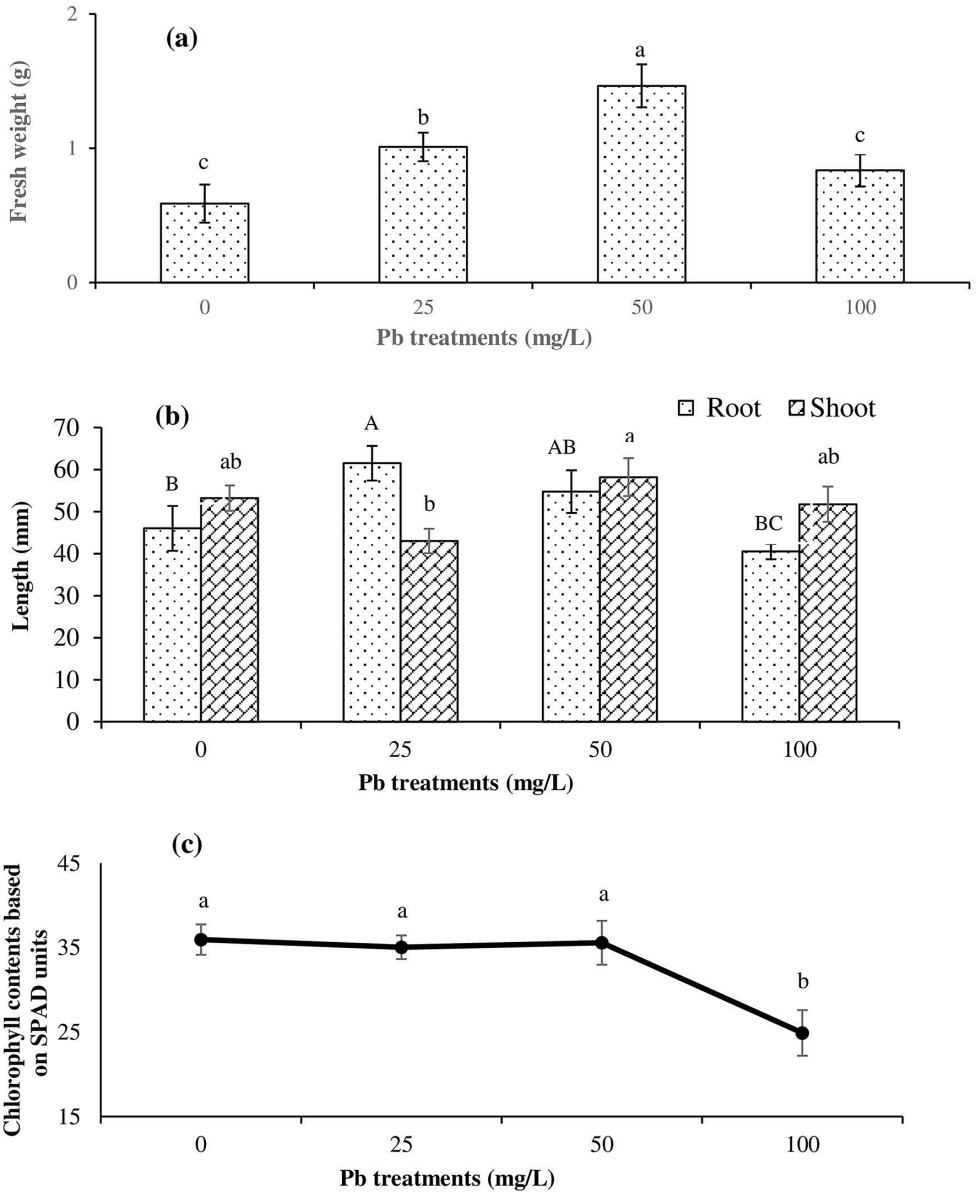
*T. qataranse* response to Pb treatments (**a**) fresh weight, (**b**) root and shoot length and, (**c**) chlorophyll contents. Means are averages from five replicates (*n* = 5) ± SEM. Within each graph, mean values with common letters are not significantly different at *p* ≤ 0.05, according to Tukey’s test. In graph (**b**) the significance among treatments are within the root or within the shoot.

Plants tend to be more susceptible to metal toxicity after germination^11^. Therefore, the evaluation of seedling growth is vital in assessing metal toxicity in plant systems. The effects of Pb concentration on total chlorophyll content, fresh weight, root, and shoot length were evaluated and the results are shown in Fig. 1. A general increase (*p* ≤ 0.05) in *T. qataranse* fresh weight was observed under 25 and 50 mg/L Pb treatments (Fig. 1a). The highest biomass accumulation (~ 1.5 g) was observed for the 50 mg/L treatment concentration, suggesting probable growth stimulation at that level. Regarding the lengths of shoots and roots, *T. qataranse* tissue response varied with increasing Pb concentration (Fig. 1b). While there was significant root length increase (*p* ≤ 0.05) for the 25 mg/L treatment and an increase for both shoot and root in the 50 mg/L treatment compared to the control, the length of shoots and roots in the 100 mg/L treatment were not significantly different from the control treatment. While no effect occurred for leaf chlorophyll content in either the 25 or 50 mg/L treatments, a significant decline occurred in the 100 mg/L treatment compared with the control treatment (Fig. 1c).

### Pb bioaccumulation in *T. qataranse*

**Figure 2:**
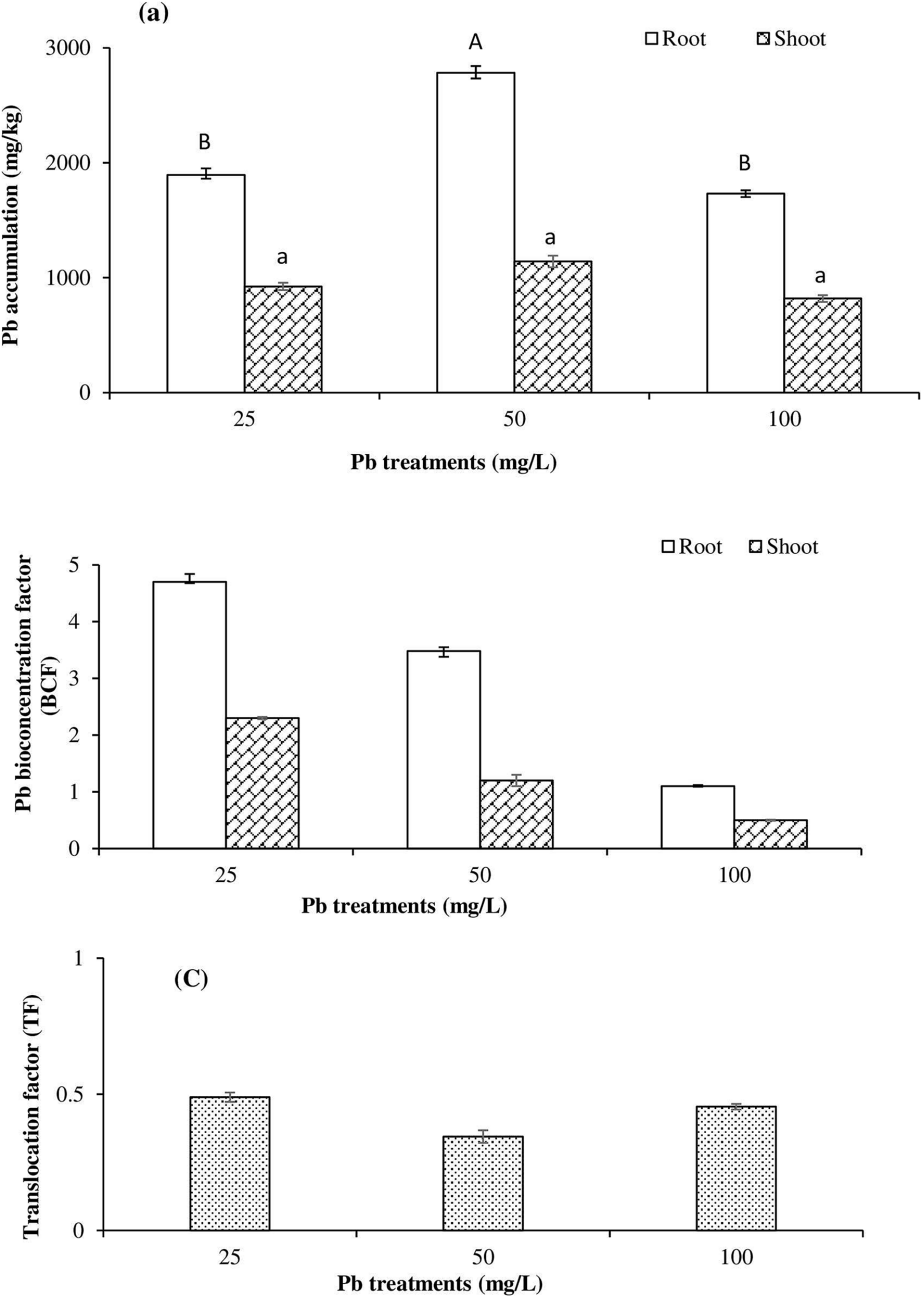
(**a**) *T. qataranse* mean root and shoot Pb accumulation. (**b**) Shoot and root Pb bioconcentration factor and (**c**) Pb translocation factor (TF). Means represent averages of three replicates (*n* = 3) ± SEM. In graph (**a**), and within root or shoot biomass mean values with common letters are not significantly different at *p* ≤ 0.05, according to Tukey’s test.

Our group previously reported Pb accumulation on *T. qataranse*^7^. However, our study was based on field conditions and in the presence of other metals. Here, for the first time, we report Pb accumulation in *T. qataranse* grown under greenhouse conditions (Fig. 2a). In the 50 mg/L Pb treatment, where the total Pb concentration in the growth medium reached 800 mg/kg at harvest, the root accumulated 2784 mg/kg of Pb, while there was a Pb concentration of 1141.6 mg/kg in the shoot. The lowest Pb accumulation, of 1732 mg/kg and 817 mg/kg Pb for root and shoot, respectively, was in the 100 mg/L treatment. At this concentration, the total Pb concentration in the growth medium reached 1600 mg/kg at harvest. The soil pH and total organic matter (TOC) were 7.35 and 1.87%, respectively. Given the neutral pH and the low percentage of organic matter in the growth medium, the effects of pH and TOC on Pb bioavailability and uptake by *T. qataranse* can be eliminated.

### Bioconcentration and translocation factor

The bioconcentration factor (BCF) and translocation factor (TF) are indices that are essential in the evaluation of metal bioaccumulation and translocation in plant tissues^12^. The BCF estimates metal concentration against concentration in the treatment medium while the TF determines whether plants translocate metals to their aerial parts. Figure 2b,c show Pb BCF and TF, respectively. In order of increasing concentration of Pb treatments, the root BCF was 4.7, 3.5, and 1.15, whereas the shoot BCF was 2.3, 1.4, and 0.5. The TF of all treatments was less than one (Fig. 2c), indicating that *T. qataranse* preferentially accumulates Pb in the root.

### Antioxidative response

**Figure 3:**
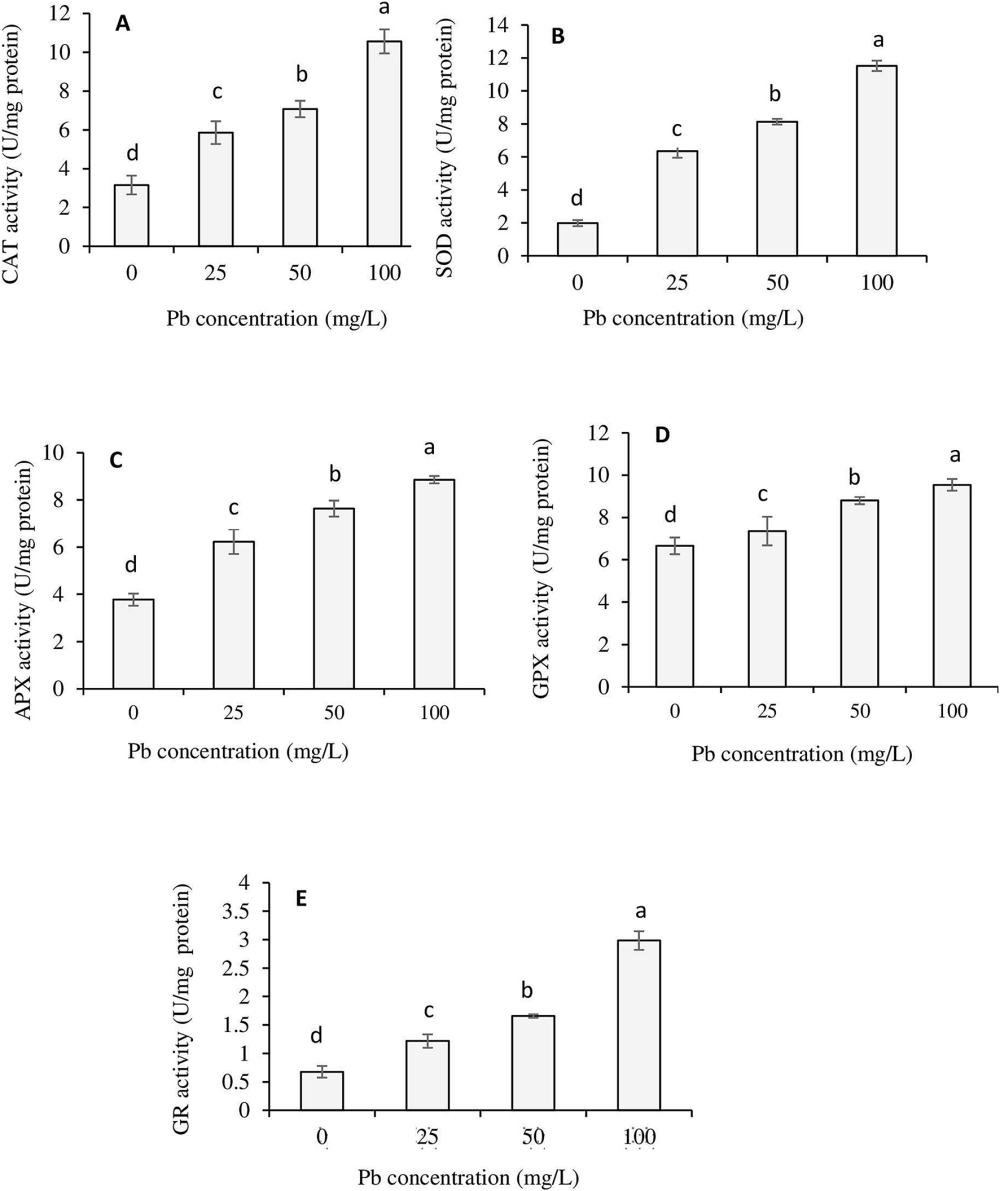
Enzymes activities in *T. qataranse* (whole plant) due to Pb stress (**A**) Catalase (CAT) (**B**) superoxide dismutase (SOD) (**C**) ascorbate peroxidase (APX) (**D**) guaiacol peroxidase (GPX), and (**E**) glutathione reductase (GR). Means represent the average of three replicates (*n* = 3) ± SEM. Within each graph, mean values with common letters are not significantly different at *p* ≤ 0.05, according to Tukey’s test.

The results of an antioxidant enzyme assay showed a concentration-dependent increase in enzyme activity in Pb-treated *T. qataranse* when compared to the control in the order SOD > CAT > GPX > APX > GR (Fig. 3a–c). This work is the first that reports on the oxidative status of *T. qataranse* due to Pb stress. Our result is consistent with the findings of Sidhu et al., 2016, where an increase in the activities of all five enzymes was observed in Pb-treated *Coronopus didymus*^4^. Similarly, increased activities of CAT and APX due to Pb treatment were reported in *Zygophyllum fabago*^16^. ROS play a critical role in redox signaling and is essential for cellular homeostasis. The enzymes analyzed in the present study are primarily responsible for the reduction of free radicals in plant systems^13^.

## Discussion

The evaluation of *T. qataranse* growth parameters suggests that Pb has no adverse effect on the plant at concentrations of less than 100 mg/L Pb. However, at 100 mg/L, the metal disturbed healthy growth and, in particular, interfered with root development. Consistent with our findings, a similar study using *Z. fabago* reported that Pb negatively affects root development^14^. The root plays a vital role in plant health and development, influencing other tissues’ response to stress conditions. Despite it being one of the most critical parameters in the assessment of plant health, a significant reduction in total chlorophyll content was observed (Fig. 1c). However, Pb toxicity symptoms (e.g., leaf chlorosis and root darkening) were not apparent across any of the treatments. Typically, Pb accumulation in plants raises the level of chlorophyllase, an enzyme that negatively affects chlorophyll. An increased level of chlorophyllase slows down photosynthesis and, therefore, affects overall growth and development. Consequently, due to slow metabolic activities, cell division is adversely affected and healthy growth is inhibited^15^.

Pb accumulates differently in plant tissue parts, especially in the root^22^. Concerning *T. qataranse* Pb accumulation, overall data across all treatments indicates that *T. qataranse* preferentially concentrates Pb in the root (up to 2,784 mg/kg)*.* Our result is consistent with the reports of many similar studies. For instance, known plant species, including *Nerium oleander* L. and *Brassica juncea*, accumulate higher Pb concentrations in their roots than other tissue parts^16^. Also, in a study involving different plants, Finster, et al.^17^ determined that the roots always accumulate more Pb than other plant parts, including the fruits, where only traces of the metal translocate the shoot. Our result is also in agreement with the work of Langley-Turnbaugh and Belanger^18^. Kumar, et al.^19^ and Pourrut, et al.^20^ conducted several critical reviews of Pb toxicity in plants and determined that several factors contribute to restricted metal translocation in plants. Of such, Casparian strip endodermis restriction is by far the most limiting for Pb. Notheless, the ability of *T. qataranse* to accumulate more than 1000 mg/kg Pb suggests that it is a Pb hyperaccumulator^21^.

Additionally, the root BCF across all treatments was higher than that of the shoot (Fig. 2b). The BCF indicates that, to some degree, *T. qataranse* sequestrate Pb from growth medium that contains up to 1600 mg/kg Pb. However, it was optimal in the 50 mg/kg treatment. At this concentration, the growth medium had up to 800 mg/kg Pb. The TF under all treatments was less than 1 (Fig. 2c), meaning that *T. qataranse* can not sufficiently transfer Pb to its aerial parts. In the current study, the restriction of Pb translocation finding is consistent with our previous report on *T. qataranse* where field samples were analyzed for various metals accumulation, including Pb^22^. Some of the factors that affect metal bioavailability and uptake include plant and metal types; metal form, concentration, and age in the soil; pH; and organic matter content. However, pH and total organic matter content are the most critical in terms of metal bioavailability and uptake of Pb. The pH significantly affects the behavior of Pb by dictating its chemical form. Metals, including Pb, are more soluble at low or near-neutral pH values. At pH > 8, metals tend to precipitate in the soil. Similarly, a high TOC limits the bioavailability of Pb^11^. In this work, the pH and TOC in the growth medium were 7.35 and 1.87%, respectively. Therefore, given the neutral pH and low TOC, their effects on Pb bioavailability and uptake by *T. qataranse* was insignificant and can be eliminated.

Various response mechanisms enable plants to withstand metal toxicity, of such, metal avoidance and uptake are the most common. Before compartmentalization, Pb is translocated to a degree that can be described by the TF^23^. Pb mainly precipitates on the root cell wall and only the free ions are transported to other parts via the xylem and phloem cells^24^. Previous works confirmed that Pb disrupts cellular homeostasis by replacing essential cations and altering metal-containing enzyme activity. In plants, the primary sources of ROS are chloroplasts, mitochondria, and peroxisomes. Pb toxicity interferes with electron transport chains in turn increasing ROS accumulation. Nearly every stage of the central dogma of plants (DNA, RNA, protein) is affected by Pb toxicity^25^.

The antioxidant system is one mechanism used by plants for protection against metal toxicity. In this study, the result of the SOD, CAT, APX, GPX, and GR assay show increased activity of all five enzymes. SOD activity was the highest, up to ten times higher than the control (0 mg/kg Pb), particularly in the root (Fig. 3a), suggesting the critical role of SOD in *T. qataranse* antioxidative defense. Having the highest enzymatic activity be in the root changes root organic constituents due to Pb complexation. This indicates that, as suggested in our previous work, as an uptake mechanism, Pb ions bind to *T. qataranse* root by complexation through cationic exchange with hydroxyl and carboxyl functional groups^7^. This is a well-established complexation mechanism of transition metals, including Pb^1^. In comparison, GR demonstrated the least activity (Fig. 3e). Such differences can be attributed to the specific roles each enzyme has in ameliorating Pb stress. Many other studies reported a similar increase in the activities of one or all the enzymes analyzed in this work following plants’ exposure to Pb; examples are increases in the activity of CAT, SOD, APX, and GPX in *Ceratophyllum demersum* L.^26^ and the cotton plant^27^; SOD, APX, GPX, and GR in *Oryza sativa* L.^28^; and APX, CAT, and GR in *Triticum aestivum*^9^. Changes in enzymatic activity account for the elimination of ROS and the improvement of stress conditions in plants. Therefore, the enhanced activities of all enzymes suggest that their role is crucial in ameliorating Pb toxicity in *T. qataranse*. Other studies support this conclusion, including Ferrer, et al.^16^ who attribute enhanced CAT and APX activities to the efficient ROS scavenging capability of *Z. fabago* exposed to Pb. In addition, Nikalje and Suprasanna^30^ reviewed several other similar studies involving halophytes . However, to the best of our knowledge, our work is the first on *T. qataranse.* In addition, primarily due to GR activity suggesting the utilization of glutathione, we can conclude that Pb detoxification in *T. qataranse* may partly involve glutathione metabolism^30^. Glutathione, which exists in either the reduced (GSH) or oxidized form (GSSG), acts as an antioxidant and chelating bioligand majorly accountable for metals detoxification. Enzymes involved in glutathione metabolism mediates detoxification Glutathione-S-transferases (GSTs) are a major phase II GSH-dependent ROS scavenging enzymes. They play significant roles in GSH conjugation with exogenous and endogenous species found during oxidative stress, including H2O2 and lipid peroxides^9,10^. Consistent with our findings, a more recent review by Kumar and Prasad^14^ discussed several other studies, some of which include the use of model species, *Arabidopsis thaliana*, and *Oryza sativa*, all of which support our findings.

It is worth mentioning that part of the discussion presented in this work is limited to the perspective of deciphering Pb tolerance and uptake mechanisms from metal translocation and plant antioxidative systems. However, both molecular and biochemical mechanisms play significant roles in toxic metals detoxification, including Pb. For instance, glutathione metabolism is known to regulates the biosynthesis of phytochelatins (PC), which bind Pb and transports it to vacuoles where detoxification can occur. Additionally, genes, such as glutamate cysteine ligae 1 (GSH1), glutamate cysteine ligae 1 (GSH2), phytochelatins synthase 1 (PCS1), and phytochelatins synthase 2 (PCS2), are actively involved in GSH-dependent PCs synthesis. Other primary and secondary metabolites that act as antioxidants, such as Tocols, flavonoids, anthocyanidins, and ascorbic acid, are essential to protecting plants against oxidative damage as well. The functions of these metabolites are well documented^8,9,20^. Further, we recognize that proteins regulate ROS signaling and the expression of such proteins changes due to Pb exposure^11^. Due to metal stress in plants, increased protein synthesis is essential in cellular metabolic processes. Mitogen-activated protein (MAP) kinase pathways regulate such processes, serving as a signaling system against oxidative stress. Signaling occurs through multiple stages of the reaction, modifying gene expression and, ultimately, protein synthesis^4^. Therefore, our future work will focus on the differential expression of proteins, particularly those that are related to stress responses, such as the heat shock protein family, due to Pb exposure in *T. qataranse*.

## Conclusion

In this study, we showed that Pb stimulates growth and accumulates in high concentration in *T. qataranse*. However, while the plant demonstrates good Pb accumulation capacity, further studies are required to ascertain its suitability for practical phytoremediation application. Additionally, our results showed that *T. qataranse* copes with high levels of Pb via the antioxidant system by synthesizing and increasing the activities of critical enzymes, CAT, SOD, APX, GPX, and GR, which are capable of reducing and manipulating free radicals. However, to sufficiently elucidate the mechanisms of Pb tolerance and uptake, further biochemical and molecular studies are imperative.

## Materials and methods

### Seedling collection and pre-treatment

Young seedlings that were approximately one week old were collected from Qatar University campus (25° 22′ 21.60″ N 51° 29′ 45.28″ E) Doha-Qatar, during October and November 2018. The young seedlings were planted in pots containing regular soil amended with peat moss in a ratio of 3:1. Before transferring the mixture into the pots, the pH and total organic content (TOC) were measured. Each young *T. qataranse* seedling and control replicate was planted into a pot containing approximately 300 g of the media for individual treatment. Before Pb treatment, the young seedlings were irrigated with distilled water for three weeks under greenhouse conditions.

### Pb treatments

A modified Hoagland nutrient with and without Pb for treatment and controls, respectively, was prepared as the base solution for seedling irrigation. For the treated plots, seedlings were irrigated with a solution prepared using lead chloride (PbCl_2_) to create varying Pb concentrations of 25, 50, and 100 mg/L Pb. Only the modified Hoagland nutrient solution (0 mg/L Pb), was used for the control plot^29^. Treatment concentrations were chosen based on Pb soil concentrations in Qatar^7^ and the hyperaccumulation threshold.

### Seedling irrigation

**Figure 4:**
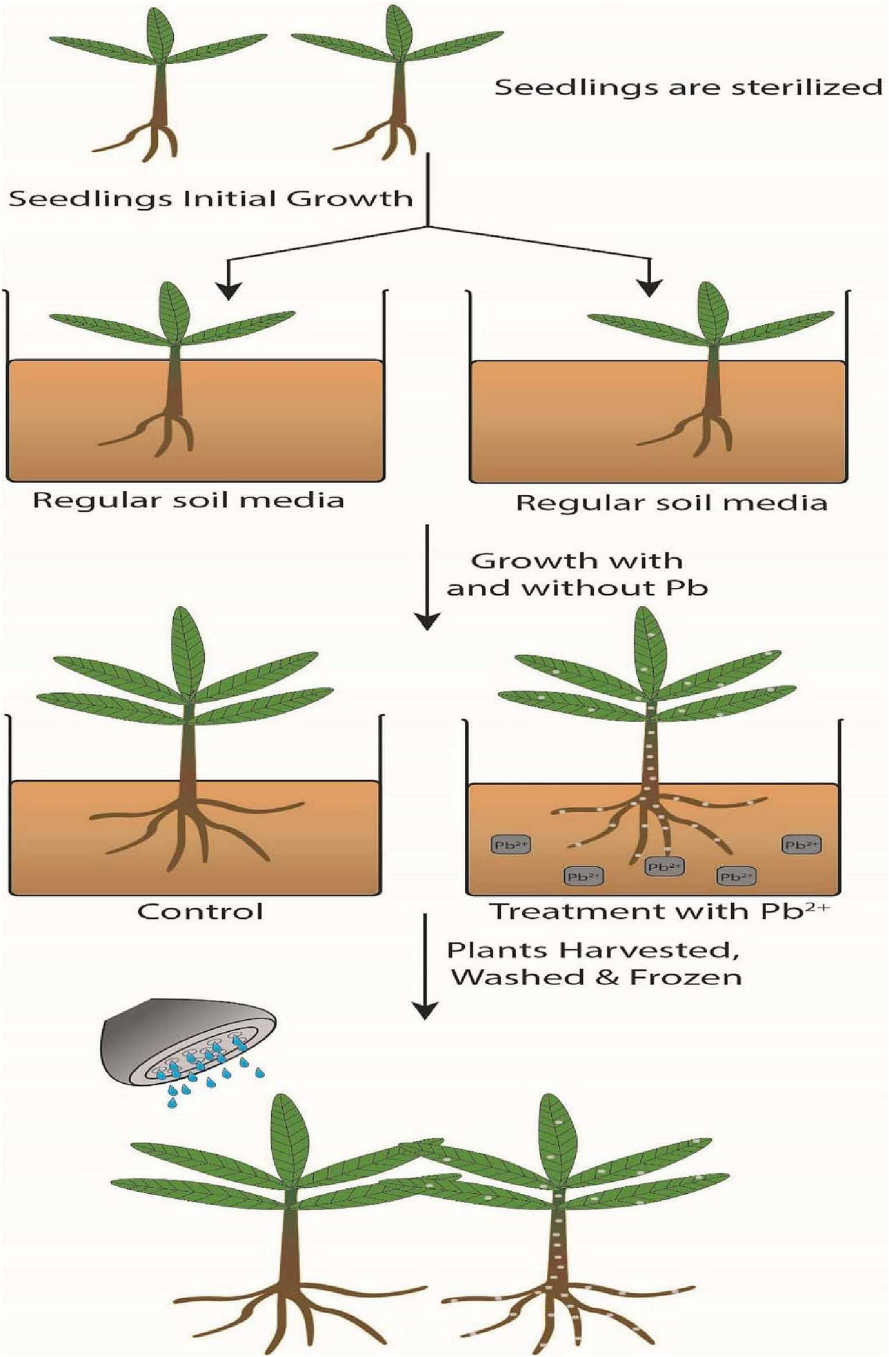
Schematic representation of summarized *T. qataranse* lead (Pb) phytotoxicity test.

For the actual experiment, three-week old seedlings of similar height and weight were selected. Five seedlings were planted (three replicates per treatment) in a randomized manner and irrigated with 200 mL of solution every 48 h for seven weeks. During irrigation, no leaching of the Pb treatment solutions occurred. After seven weeks, growth parameters were evaluated as described in Usman, et al.^30^. A schematic representation of the experimental set up is shown in Fig. 4.

### Pb quantitation using inductively coupled plasma optical emission spectroscopy

Harvested plant biomass was prepared by digestion in nitric acid and hydrogen peroxide before analysis. This was accomplished using a large capacity Environmental Express SC154 HotBlock® digestion system at alternating temperatures until solutions were clear. Standard linear curves were obtained using five different concentrations from 1.0 to 500 µg/L. One standard and quality check analysis was performed and a good correlation coefficient of > 0.999 was obtained. Quantitation of Pb accumulation in *T. qataranse* tissues was performed using inductively coupled plasma optical emission spectroscopy (ICP-OES). All samples were in three replicates and were measured against Standard Reference Materials (SRMs): soil 2709a and apple leaves 1515^34^.

### Bioconcentration and translocation factor

The bioconcentration factor (BCF; a ratio of metal concentration in plant tissues to metal concentration in the soil) and translocation factor (TF; a ratio of metal concentration in the shoot to metal concentration in the root) were evaluated, as previously reported in Usman et al.^7^.

### Antioxidant enzymes assay

Enzyme extraction followed the method of Mishra et al.^26^. Briefly, 0.2 g of a fresh tissue sample was homogenized in an ice-cooled mortar using 5 mL 10 mM potassium phosphate buffer (pH 7.0), 1% (w/v) polyvinylpyrrolidone, and 0.1 mM Ethylenediaminetetraacetic acid (EDTA). This was followed by a centrifugation of the homogenate at 15,000*g* for 15 min at 4 °C. Enzymatic activities were determined and expressed as Unit/mg of protein.

To measure SOD (EC 1.15.1.1) activity, nitroblue tetrazolium (NBT) photochemical reduction inhibition was determined by Beauchamp and Fridovich^31^. A total of 3 mL of assay mixture containing 40 mM phosphate buffer (pH 7.8), 13 mM methionine, 75 µM NBT, 2 µM riboflavin, 0.1 mM EDTA, and an aliquot of enzyme extract was used for the reaction. After 15 min under illumination, absorbance was recorded at 560 nm. The non-illuminated mixture was used as the control.

The CAT (EC 1.11.1.6) assay was performed according to Zhang et al.^32^. A total reaction mixture of 3 mL was prepared. The reaction mixture was comprised of 50 mM sodium phosphate buffer (pH 7.0), 20 mM H_2_O_2,_ and an extract of the enzyme. Changes in the absorbance of the solution were measured at 240 nm for 4 min.

The determination of APX (EC 1.11.1.7) activity followed the ascorbic acid oxidation rate, as described in Nakano and Asada^33^. Briefly, 3 mL of reaction mixture containing 50 mM phosphate buffer (pH 7.0), 0.1 mM H_2_O_2_, 0.5 mM sodium ascorbate, 0.1 mM EDTA, and the enzyme extract were used. The changes in absorbance were monitored at 290 nm.

GPX (EC 1.11.1.9) activity following guaiacol oxidation was measured by measuring an increase in absorbance at 470 nm as described in Egley et al.^34^. The reaction mixture consisted of 10 mL 1% guaiacol (v/v), 10 mL 0.3% H_2_O_2,_ 80 mL 50 mM phosphate buffer (pH 6.6), and the enzyme extract.

GR activity was determined by measuring absorbance at 340 nm due to NADPH oxidation, following the method of Rao et al.^35^. The reaction mixture contained 100 mM potassium phosphate buffer (pH 7.8), 2 mM EDTA, 0.2 mM NADPH, 0.5 mM GSSG, and the enzyme extract.

### Statistical analysis

Analysis of variance (ANOVA) was performed to study the significant differences in growth changes between the Pb-treated plants and control plants, at *p* ≤ 0.05. ANOVA was also performed to study the significance of Pb treatments on oxidative stress enzyme activities. The Shapiro–Wilk test was used to test the normality of the data and Tukey’s test was used to separate the means at *p* ≤ 0.05.
